# Precise Construction and Growth of Submillimeter Two-Dimensional WSe_2_ and MoSe_2_ Monolayers

**DOI:** 10.3390/ma16134795

**Published:** 2023-07-03

**Authors:** Yuqing Li, Yuyan Zhao, Xiaoqian Wang, Wanli Liu, Jiazhen He, Xuemin Luo, Jinfeng Liu, Yong Liu

**Affiliations:** 1 International School of Materials Science and Engineering (ISMSE), State Key Laboratory of Advanced Technology for Materials Synthesis and Processing, Wuhan University of Technology, Wuhan 430070, China; lyqlosyimi@163.com (Y.L.); 303568@whut.edu.cn (X.W.); liuwani97@163.com (W.L.);; 2 Southwest Institute of Technical Physics, Chengdu 610041, China; yuyan_zhao0514@163.com

**Keywords:** two-dimensional materials, transition metal dichalcogenides, chemical vapor deposition

## Abstract

Currently, as shown by large-scale research on two-dimensional materials in the field of nanoelectronics and catalysis, the construction of large-area two-dimensional materials is crucial for the development of devices and their application in photovoltaics, sensing, optoelectronics, and energy generation/storage. Here, using atmospheric-pressure chemical vapor deposition, we developed a method to regulate growth conditions according to the growth mechanism for WSe_2_ and MoSe_2_ materials. By accurately controlling the hydrogen flux within the range of 1 sccm and the distance between the precursor and the substrate, we obtained large-size films of single atomic layers with thicknesses of only about 1 nm. When growing the samples, we could not only obtain a 100 percent proportion of samples with the same shape, but the samples could also be glued into pieces of 700 μm and above in size, changing the shape and making it possible to reach the millimeter/submillimeter level visible to the naked eye. Our method is an effective method for the growth of large-area films with universal applicability.

## 1. Introduction

The atomic-level thinness of two-dimensional materials brings unique electronic and optical properties. As an important type of these materials, transition metal dichalcogenide (TMD) materials are of interest because of their high carrier mobility, chemical stability, tunable bandgap, light–matter interaction, bipolar properties, high on/off ratio, and high electro/photocatalytic activity [1,2]. The theoretical limiting mobility of monolayer MoS_2_ and WS_2_ phonons at room temperature is about 410 cm^2^·V^−1^·s^−1^ [3] and 1100 cm^2^·V^−1^·s^−1^ [4], respectively, which is a clear advantage of two-dimensional materials for transistor size-scaling. Eight different switching states can be realized in electrically tunable homojunction devices based on bipolar WSe_2_ materials. This helps to build reconfigurable multifunctional logic circuits with high area efficiency [5]. In addition, TMD materials reveal dimensionally relevant quantum phenomena [6,7,8,9], such as two-dimensional superconductivity, magnetism, topologically protected states, and quantum transport [10,11,12,13,14,15,16]. Certain unconventional phases of MoS_2_ and WSe_2_ (octahedral, 1T, and 1T’) are semimetals with metal charge transport. They have a wide range of applications in energy storage and conversion [17] characterized by their use as catalysts for hydrogen evolution reactions (HERs) [18]. The narrow bandgap of two-dimensional materials allows them to exhibit photocatalytic properties in the solar spectral region [19]. The combination of monolayer MoS_2_/monolayer WSe_2_ and other light-harvesting materials to form Si/2D or “all 2D” heterojunction solar cells [20] can achieve efficiencies up to 2.56% [21], and it is clear that stacking TMD materials using van der Waals forces for application in heterogeneous or homogeneous structures will lead to new phenomena and derived device concepts [22,23,24,25], making it possible to achieve high electrocatalytic activity [1]. However, the difficulty of synthesizing large-area monolayers and few-layer films hinders the development of technology associated with TMDs, limiting their application in two-dimensional crystal production and complicating further processing (e.g., fabrication of heterostructures). The technology for the synthesis of large-size films is critical to apply their excellent electrical, optical, and chemical properties in a wide range of electronic, optoelectronic, and sensor devices [26,27,28,29,30,31].

Two commonly used preparation methods are mechanical exfoliation and vapor phase deposition. From the first isolation of graphene, mechanical exfoliation has been the key to providing high-quality two-dimensional materials. Despite improvements [32,33], the problems that can be solved in terms of yield, lateral dimensions, and contamination are still limited. It is difficult to avoid damage, which results in great obstacles in the preparation process. Currently, chemical vapor deposition (CVD) is the most widely used method [34,35,36], making it possible to grow single-molecule films with high optical and electronic quality and achieve an optimal balance between material quality, area coverage, and scalability potential. However, compared to other low-dimensional materials for device applications, such as chalcogenide solar cells, the size of two-dimensional materials is still far from being standardized for industrial production and applications. In recent years, two-dimensional materials have been grown down to the millimeter scale by changing the growth substrate. For example, WS_2_ thin films have been successfully synthesized on c-plane (0001) sapphire through strain engineering [37,38]. Wafer-scale MoS_2_ films can be obtained through the epitaxial growth of single crystals using commercial gold foil substrates [39]. Placing Au layers in contact with two-dimensional-material crystals and heating them can yield large-area monolayers of MoS_2_ and WS_2_, but the cost is high and it is difficult to guarantee material quality [40]. Alternatively, the precursors can be improved by producing inch-scale monolayers of MoS_2_ using pre-deposited MoO_3_ films in a sulfur vapor atmosphere [41] or metal–organic CVD (MOCVD) systems can be used with gas-phase organometallic compounds as metal precursors instead of conventional metal oxide powders to grow wafer-scale TMD films [42]. However, the small domain size makes it difficult to grow highly crystalline films. The growing conditions are generally harsh and mass production is difficult [43,44,45,46]. Combining the above studies (we surveyed representative studies on size and thickness in the literature from recent years, and the details are shown in Table S1 in the Supplementary Material), we finally chose an atmospheric-pressure chemical vapor deposition method that has been extended to industrial production and allows precise control of shape according to the conditions required for growth, making it possible to obtain excellent samples. TMD flakes can overcome the problem of difficult scale-up changes and grow stably and repeatedly into continuous films that can be merged.

Here, we report a method for synthesizing large-size two-dimensional XSe_2_ (X = W, Mo) flakes directly on Si/SiO_2_ substrates using an atmospheric-pressure chemical vapor deposition (APCVD) system that involves precisely controlling the conditions (the precursor substrate spacing and hydrogen content (to one decimal place)) and thus tuning the γSe/γX (X = W, Mo) values to achieve precise construction of two-dimensional WSe_2_ and MoSe_2_ of different shapes at the millimeter/submillimeter level. The thickness, morphology, and composition of the XSe_2_ (X = W, Mo) thin films were characterized using optical microscopy (OM), AFM, Raman spectroscopy, and XPS. The results showed that we could obtain both fixed-shaped XSe_2_ (X = W, Mo) flakes and large films at the millimeter/submillimeter level, providing a more straightforward method for subsequent heterojunction construction or device applications with more convenient operation under low-magnification optical microscopy conditions.

## 2. Materials and Methods

### 2.1. Materials

WO_3_ (Sigma-Aldrich, St. Louis, MO, USA, 99.99%) and MoO_3_ (Sigma-Aldrich, 99.9%)/KCl (Sigma-Aldrich, 99.99%) mixtures were used as growth precursors, and Se (Alfa Aesar, Haverhill, MA, USA, 99.9%), acetone (Sinopharm Chemical Reagent, Shanghai, China, AR), and anhydrous ethanol (Sinopharm Chemical Reagent, AR), respectively, were used to clean the Si/SiO_2_ substrates of the grown materials (Hefei Kejing Materials Technology Co., Hefei, China, AR). Ar was used as the transport carrier gas, and H_2_/Ar (10% H_2_ content) was used as the reaction gas (Wuhan Newradar Special Gas Co., Wuhan, China, 99.999%).

### 2.2. Growth Preparation and Processes

Using the solid precursors MoO_3_ and WO_3_, we synthesized large monolayers of MoSe_2_ and WSe_2_ on 300 nm Si/SiO_2_ substrates by using atmospheric-pressure chemical vapor deposition in a single-temperature-zone tube furnace with a tube diameter of 1 inch. Materials grown on Si/SiO_2_ substrates are easier to distinguish from those grown on sapphire and fluoride mica substrates. The Si/SiO_2_ substrates cut to the size of 8 mm × 10 mm were successively placed in acetone, anhydrous ethanol, and deionized water for ultrasonic processing to remove oil residues and organic impurities on the surface, all of which lasted more than 15 min. For the growth of XSe_2_ (X = W, Mo), the cleaning step with acetone is indispensable. Organic impurities, which are difficult to remove even at high temperatures, cause the two-dimensional material to nucleate at the impurities, generating extremely thick bulk particles. After being cleaned, the wafers were sealed in deionized water to prevent ash fall, and the edges of the substrates could be taken out of the water with tweezers during the experiment; alternatively, the wafers could be dried by blowing them dry with nitrogen for a clean and optimal growth environment.

Regarding the synthesis of MoSe_2_, the two powders were mixed uniformly before growth with the aid of KCl. The clean substrate was placed vertically against the end of the quartz boat where the oxide precursor was placed, and the distance to the precursor (150 mg MoO_3_ with 300 mg KCl or 750 mg WO_3_) was adjusted with a gradient of 0.5 to between 5 and 20 mm. Finally, the quartz boat containing the precursor powder was moved to the center of the heating zone of the tube furnace for heating. The quartz boat containing the Se powder was placed at a distance of about 16 cm from the central heating zone where the temperature was about 270 °C, just enough to sublimate the Se powder.

After the experiment started, 300 sccm of high-purity Ar (99.999%) was first introduced. The experiment was continued for half an hour or more to exhaust the air in spite of this addition so that the growth was not disturbed by O_2_, water vapor, or other factors. Then, the Ar flow rate was reduced to 100 sccm and the temperature rise was initiated. The furnace temperature was ramped up to 950 °C over 60 min for the growth of WSe_2_ (700 °C over 45 min for the growth of MoSe_2_), and then the Ar gas was turned off and a mixture of H_2_ and Ar (99.999%) with a H_2_ content of 10% was introduced, regulated with a gradient of 0.1 between 32 and 33 sccm, and held constant for 10 min. During this time, Se vapor was transported to the growth substrate and chemically reacted with the reduced oxide precursor XO_3−x_(X = W, Mo), which eventually dispersed on the substrate and formed XSe_2_ (X = W, Mo) nucleation points with different densities. After the end of the holding period, the procedure was stopped, the H_2_/Ar was turned off, the 100 sccm passage of high-purity Ar gas was continued, and the samples were allowed to cool naturally to room temperature to obtain substrates grown with two-dimensional XSe_2_ (X = W, Mo).

### 2.3. Characterization

AFM images were obtained using a Bruker Dimension FastScan AFM (Billerica, MA, USA) in knockdown mode. Optical images were obtained using a Sunwoo RX50M microscope (Yuyao, China). Transmission electron microscopy was performed with a 200 kV Talos F200S transmission electron microscope. Room-temperature Raman and PL spectra were recorded with a Horiba Raman microscope (Irvine, CA, USA) with 532 nm laser excitation. The X-ray photoelectron spectroscopy (XPS) of the samples was carried out using a Thermo Scientific Kα XPS spectrometer (Waltham, MA, USA) equipped with a monochromatic Al-Kα X-ray source.

## 3. Results and Discussion

**Figure 1:**
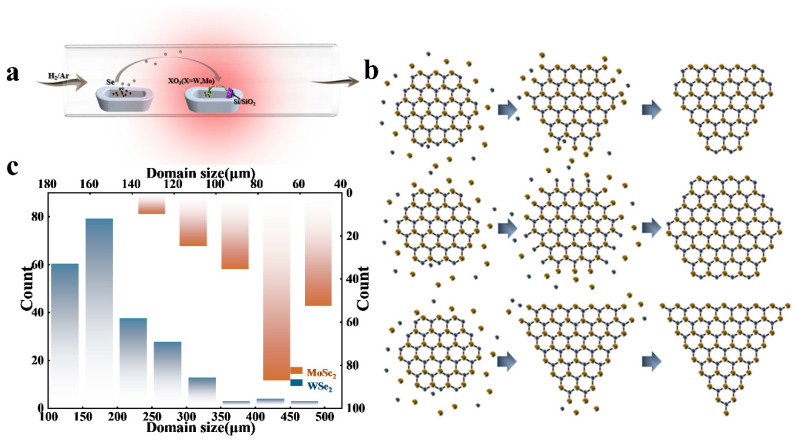
Synthesis and growth of WSe_2_ and MoSe_2_ crystals. (**a**) Improved CVD process scheme for the synthesis of triangular WSe_2_ and hexagonal MoSe_2_ crystals on SiO_2_/Si substrates. (**b**) The growth process for XSe_2_ (X = W, Mo) with different morphologies. (**c**) Size distribution of XSe_2_ (X = W, Mo) crystals.

We conducted experiments using the chemical vapor phase growth system shown in Figure 1a, which utilizes a single-temperature-zone small-diameter tube furnace for heating to minimize the effect of airflow perturbations on nucleation point deposition. The oxide precursors were placed in the center of the heating zone along with the substrate, and Se was placed at the edge of the heating zone to take advantage of the residual temperature sublimation. Carrier gas was passed in from upstream to transport the Se powder above the heating center. After growing at a fixed temperature (950 °C for WSe_2_ and 700 °C for MoSe_2_) for 10 min, the XSe_2_ (X = W, Mo) material was deposited and dispersed on the substrate surface. During most of the growth process, the substrate was placed with the polished face facing up or down [45,47,48,49]. However, the deposition density of the nucleation points was relatively limited with the parallel flow of carrier gas to the substrate surface, whether facing up or down, making it difficult to deposit uniformly. However, the distance between the substrate and the precursor itself determines the nucleation density on the substrate, and it is one of the factors worth exploring. For a single-temperature-zone tube furnace, the regional temperature is relatively concentrated, and the distance between the substrate and the precursor only needs to be slightly adjusted to affect the experimental results (in Figures S1 and S2 in the Supplementary Material, we show part of the parameter modulation process). Therefore, unlike in previous work, we leant the substrate vertically against one end of the porcelain vessel to increase the contact area for the precursor vapor and the substrate. This was beneficial for conveniently adjusting the distance between the substrate and the precursor.

**Figure 2:**
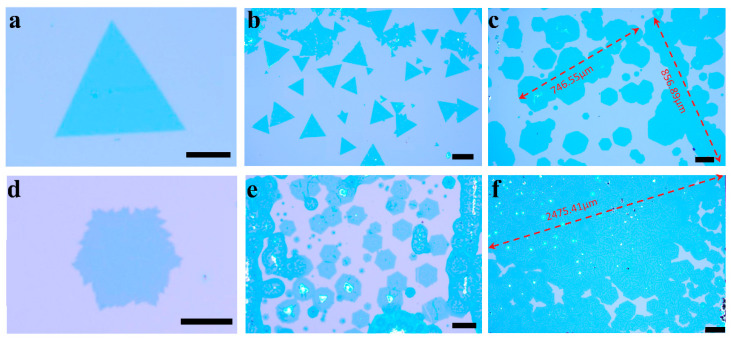
Optical images of XSe_2_ (X = W, Mo) crystals grown on SiO_2_/Si substrates. (**a**,**b**) High-magnification and low-magnification optical microscopy images of triangular WSe_2_ films, and (**c**) optical microscopy images of large coalesced monolayer hexagonal WSe_2_ films. (**d**,**e**) High-magnification and low-magnification optical microscope images of hexagonal MoSe_2_ films, and (**f**) optical microscope images of large coalesced monolayer hexagonal MoSe_2_ films. (**a**,**d**,**e**) Scale bars are 50 μm; (**b**,**c**) scale bars are 100 μm; (**f**) scale bar is 200 μm.

In the first stage of the CVD growth process, small amounts of oxide precursors and Se (Tm = 220 °C) are in the gas phase during the increase to the final temperature. This leads to a weakly reducing environment and partial reduction of XO_3_ (X = W, Mo) to XO_3−x_Se_y_ (X = W, Mo) molecular clusters, which then condense into nanoparticles (nucleation points) on the SiO_2_ substrate. As the temperature of the CVD furnace core continues to increase, the final growth temperature reaches 950 °C, leading to a transition to a moderate Se atmosphere. This leads to a complete reduction of the oxide precursors and a complete transformation of XO_3_ (X = W, Mo) to XSe_2_ (X = W, Mo) in the gas phase. During the growth process, we found that the two-dimensional XSe_2_ (X = W, Mo) material grew into different shapes, such as truncated triangles, hexagons, and square triangles, as shown in Figure 2 and Figure S3.

The competing edge free energies and the local ratio of X: Se atoms are the fundamental reasons for the formation of two-dimensional XSe_2_ (X = W, Mo) sheets with different shapes, as shown in Figure 1b. According to the simple Wulff definition, the linear growth rate of a given crystal plane group is proportional to the specific surface free energy of that group at isothermal constant capacity, so the shape of XSe_2_ (X = W, Mo) can be interpreted as the relative edge of the Se edge (γSe) and the X (X = W, Mo) edge (γX) as a function of the free energy. Competing nucleation site reactions at the XSe_2_ (X = W, Mo) edge determine the shape of XSe_2_ (X = W, Mo) crystals. With low H_2_ flux, the production of XO_3−x_ (X = W, Mo) is small relative to the Se vapor, so Se reacts with and consumes the X (X = W, Mo) edge. XO_3−x_ (X = W, Mo) selectively deposits X (X = W, Mo) atoms at the Se edge and the X (X = W, Mo) edge. The X (X = W, Mo) side reacts faster than the Se side. Therefore, continuous fast growth at the X (X = W, Mo) edges will cause the X (X = W, Mo) edges to disappear, resulting in the formation of triangular XSe_2_ (X = W, Mo) crystals with three Se edges. With increasing H_2_ concentration, the volatility of XO_3−X_ (X = W, Mo) increases, the reaction of the Se edges is enhanced, and the growth of the X(X = W, Mo) edges is correspondingly weakened [50,51,52]. When γSe/γX(X = W, Mo) > 2 or γSe/γX(X = W, Mo) < 0.5, triangular crystals appear, and when 0.5 < γSe/γX(X = W, Mo) < 2, hexagonal crystals are produced; the truncated triangles are the transition states where the two are interconverted. We counted the sizes of 222 of the triangular WSe_2_ thin-film materials grown and 212 of the hexagonal MoSe_2_ ones and plotted the size distribution statistics, as shown in Figure 1c. The area for WSe_2_ was mainly concentrated between 150 and 200 μm, while the area for MoSe_2_ was mostly concentrated between 60 and 80 μm; the overall area for WSe_2_ was more than twice that for MoSe_2_. Obviously, in our work, independent-discontinuity large-area films generally showed a triangular shape.

To investigate the difference between the two shapes, we selectively grew triangular WSe_2_ and hexagonal MoSe_2_. How to obtain a specific shape for a sample was also one of the focuses of our study. Figure 2 shows our optical microscopy images of XSe_2_ (X = W, Mo) obtained under precisely controlled conditions. Figure 2a,d are the high-magnification-condition images, while Figure 2b,e are the lower-magnification-condition images. As we can see from these four graphs, we grew 100% triangular WSe_2_ with hexagonal MoSe_2_. Throughout the experiment, only the H_2_ flow rate and the substrate–reactant spacing were adjusted. In this case, the H_2_/Ar mixture with 10% H_2_ content was set to between 32 and 33 sccm, with an exact gradient increase of 0.1; the substrate and reactant spacing was set from 5 to 20 mm to produce the optimal growth conditions. In our experiments, the hydrogen flow rate for growing triangles was less than that for hexagons, while the flow demand for the large coalesced monolayer films grown was in between the two (Figure 2c,f), as shown in Table 1, where the data displayed are the H_2_/Ar flow rate/precursor–substrate distance.

The tuning of γSe/γX values in the 0.5–2 range can be achieved by controlling the H_2_ flux to produce appropriate WO_3−x_ and Se fluxes. The hydrogen flux determines the shape of the grown film, and as the partial pressure of H_2_ increases, the triangular crystals transform into hexagonal shapes. As the partial pressure of H_2_ increases, the diffusive growth of the edges is limited after the hexagonal crystal size increases to a certain level [53], resulting in a nonoptimal structure with jagged edges and some folded corners, indicating a state of coexistence for the Mo and Se atoms, as shown in Figure 2d. Usually, hexagonal growth is characterized by different growth rates at the edges due to insufficient hydrogen flux, resulting in irregular hexagons with only one or two random 120° angles. In contrast, to develop a regular ortho-hexagonal shape, the control of the hydrogen flow rate is significant. We mainly regulated the precursor–substrate spacing and hydrogen content in our experiments. Compared to a large tube diameter, using a single-temperature-zone tube furnace with a 1-inch tube diameter avoids many unnecessary problems, such as gas flow perturbation and nonuniform temperature distribution. We regulated the spacing and hydrogen flow rate to one decimal place. Any slight change can result in very different morphologies for the products grown at high temperatures. For growth regulation, precision is essential.

Interestingly, in the process of adjusting the hydrogen partial pressure and the distance between the precursor and the substrate, we experimentally obtained hexagonal WSe_2_ (similarly to those reported previously [52]), and, apparently, they are easier to merge than triangles, as they had dimensions that already reached up to the millimeter level, as shown in Figure 2c. We obtained many such samples.

In the experiments, the hydrogen flux and precursor and substrate spacing together determined the distribution density of the shaped nucleation sites and the final growth morphology of the film. Hexagons were easier to merge, but the triangle area was generally larger due to the lower hydrogen partial pressure and slightly more distant spacing, which resulted in a larger area for triangles or truncated triangles with some kind of atom as the cutoff edges. In contrast, the dense distribution of the nucleation points with growth into hexagonal shapes made it easier to grow coalesced monolayers hexagonally. The combined effect of the hydrogen partial pressure, spacing, and halogen salts led to more hexagonally shaped nucleation points. Excess hydrogen atoms are also more likely to replace selenium atoms and form corrosion points [54], as shown in Figure 2e.

Moreover, if the film is to be used to construct a heterojunction, the regular hexagon can be positioned more accurately or a defined rotation angle can be employed. Also, our precise tuning made the MoSe_2_ increase somewhat with the area of the growing substrate. This is a very favorable trend for industrialization. However, the increase in the area means an increase in the number of nucleation sites, which may mean that the film is not clean enough to distribute some islands of crystal particles on it, as shown in Figure 2f, where some white dots (i.e., bulk MoSe_2_) can be seen. This indicates that there are still growth conditions that need to be explored. By overcoming this, it will be possible to obtain higher-quality thin-film samples under specific conditions.

In addition to continuous large-area films, we included the effect of ambient atmospheric pressure to grow bilayers of WSe_2_ with a cutoff triangular shape that could be directly observed by the naked eye (Figure S3). With chemical vapor deposition methods, the factors that determine the growth of two-dimensional materials are very complicated; many experiments have taken into account the effect of pressure and thus employed low-pressure growth [55,56,57]. The anisotropic stress pressure from the medium varies with the environment and affects the sample. Low pressure reduces the perturbation from the gas flow, thus reducing the interference from the carrier gas in the size and distribution range of the substrate nucleation points; this is beneficial for the growth of large film areas. Our current method can be used to grow samples with large and reproducible sizes at atmospheric pressure. In the future, reducing the air pressure while keeping other conditions unchanged may contribute to further increasing the size.

**Figure 3:**
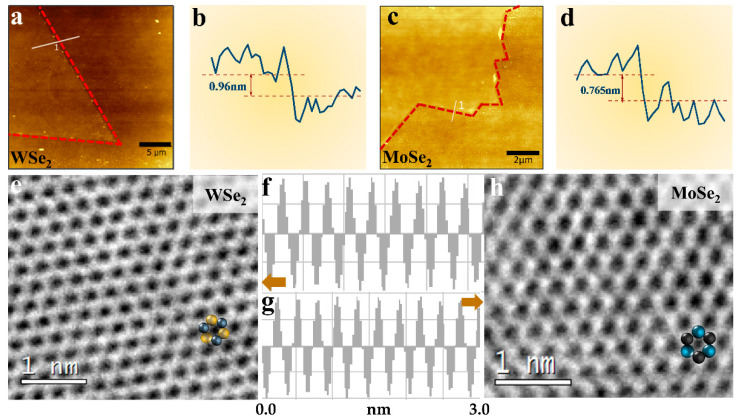
Thickness and crystal quality of XSe_2_ (X = W, Mo). (**a,b**) AFM image of WSe_2_ and its thickness, (**c,d**) AFM image of MoSe_2_ and its thickness, (**e,f**) high-resolution TEM image of WSe_2_ and its lattice spacing, (**g,h**) high-resolution TEM image of MoSe_2_ and its lattice spacing; insets show electron diffraction images with a scale of 1 nm. The arrows in (**f**) represent (**f**) in relation to figure (**e**), and the arrows in (**g**) represent (**g**) in relation to (**h**).

In addition to the more visual optical microscope image analysis, we performed morphological thickness analysis and microscopic characterization of the grown samples. Figure 3a,c show the AFM images of triangular WSe_2_ and hexagonal MoSe_2_. The film surfaces showed good homogeneity. The thicknesses of 0.96 nm and 0.765 nm for the triangles and hexagons were obtained using software scribing measurements (Figure 3b,d), which generally agreed with those reported in the literature, demonstrating the monolayer nature of the resulting samples. To further characterize the crystal quality and atomic structure of XSe_2_ (X = W, Mo), we used the high-resolution transmission electron microscopy (HRTEM) technique. The transmission electron microscopy specimens of the two-dimensional materials shown in Figure 3e,h were made using a polymer-assisted method. A thin polymer film, usually poly (methyl methacrylate), was spin-coated on the substrate of the grown target sample, which was dried and placed in a hot alkaline solution until the SiO_2_ layer was etched to release the polymer/monolayer film so that it could be applied to the TEM-specific copper mesh. The polymer was then dissolved in an organic solvent. Figure 3e shows the atomic-scale HRTEM image of the WSe_2_ sheet, and Figure 3h shows the HRTEM image of MoSe_2_. The ordered hexagonal atomic lattice indicated the high quality and monolayer nature of the XSe_2_ (X = W, Mo) flake; its six-membered-ring atomic model is shown in the lower right corner, where black indicates selenium atoms and green (blue) indicates W(Mo) atoms. The FFT spectra (Figure 3e,h, inset) demonstrated its single-crystal nature. The software measurements showed lattice spacings of 0.275 nm and 0.286 nm (Figure 3f corresponds to Figure 3e,g, which correspond to Figure 3h), which generally agree with the lattice spacings for WSe_2_ and MoSe_2_ reported in related work.

**Figure 4:**
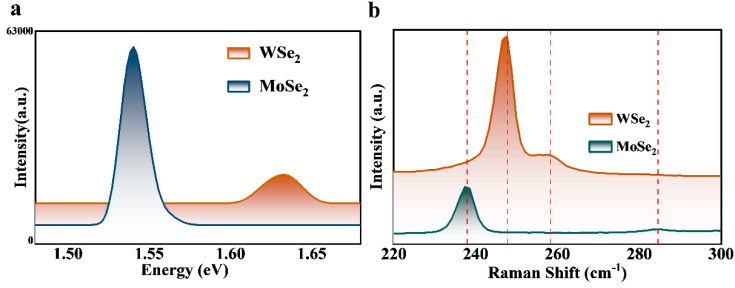
(**a**) Emission spectra for triangular WSe_2_ and hexagonal MoSe_2_. (**b**) Raman patterns for triangular WSe_2_ and hexagonal MoSe_2_. The vertical dashed lines indicates the position of the Raman peak.

Figure 4a,b show the results of our photoluminescence and Raman spectroscopy tests with triangular WSe_2_ and hexagonal MoSe_2_ using a 532 nm laser. The room temperature photoluminescence (PL) mapping (1.42 eV–1.76 eV) showed that the WSe_2_ and MoSe_2_ monoatomic layers exhibited strong PL emissions, with their prominent emission peaks being located at 1.63 eV and 1.54 eV, consistent with the direct bandgap width corresponding to the monolayer XSe_2_ (X = W, Mo). The Raman spectra clearly showed the CVD-prepared monolayer WSe_2_ films at 258.37 cm^−1^ and 247.63 cm^−1^ and the monolayer MoSe_2_ films at 237.94 cm^−1^ and 284.66 cm^−1^, respectively. The in-plane A_1g_-mode and E^1^_2g_-mode characteristic resonance peaks were shown, proving the target products’ synthesis.

**Figure 5:**
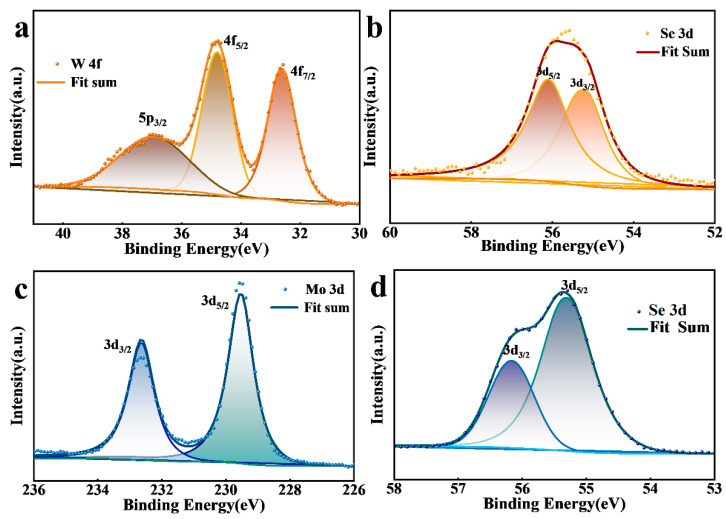
XPS high-resolution spectra for triangular WSe_2_ and hexagonal MoSe_2_ films. (**a**) W 4f, (**c**) Mo 3d, and (**b**,**d**) Se 3d.

We compared the XPS maps with other growth morphologies for XSe_2_ (X = W, Mo). As shown in Figure 5a, the W^4+^ oxidation state corresponded to the 4f_5/2_ (34.79 eV) and 4f_7/2_ (32.64 eV) peaks, respectively, and there was a W 5p_3/2_ low-intensity peak at 36.95 eV, which may have been due to the fact that a portion of the incompletely reduced tungsten oxide was still present. Similarly, the 3d_3/2_ and 3d_5/2_ peaks of Mo at 232.53 eV and 229.44 eV, respectively, indicated the Mo^4+^ oxidation state, as shown in Figure 5c. WSe_2_ corresponded to binding energies of 55.26 eV and 56.11 eV for Se 3d_5/2_ and Se 3d_3/2_, respectively, as shown in Figure 5b. MoSe_2_, on the other hand, corresponded to Se binding energy peaks of 55.31 eV and 56.16 eV, as shown in Figure 5d. Compared to previous reports, the binding energies of the different elements of the triangular WSe_2_ and hexagonal MoSe_2_ were found to be within the error range [52,58]. This result indicated that the samples had high chemical purity and the change in shape did not affect their properties. Investigation of the scanning XPS full spectra (Figure S4) indicated the direct synthesis of XSe_2_ (X = W, Mo) crystals on SiO_2_ substrates.

## 4. Conclusions

In summary, we prepared large-area two-dimensional XSe_2_ (X = W, Mo) crystals using atmospheric-pressure chemical vapor deposition with more precise control of the H_2_ flow rate and substrate distance (one decimal place) than previously reported in the literature. The high-quality morphology, structure, and composition of the grown films were confirmed by a series of characterizations (atomic force microscopy, high-resolution transmission electron microscopy, photoluminescence spectroscopy, Raman spectroscopy, and X-ray photoelectron spectroscopy). We achieved a high range of 100 percent for the desired growth shape ratio, indicating that the conditions we implemented are universal and suitable for large-scale growth of two-dimensional materials. At the same time, we found that hexagonal materials were easier to merge and grow into millimeter/submillimeter-size materials than triangular shapes. The large hydrogen flux allowed the hexagonal edge atoms to compete for growth, enabling two nucleation points at the right distance to eventually grow and converge to form a large-area single-layer film with an unbounded shape. Moreover, combining both factors and environmental influences, we achieved the growth of double-layered truncated WSe_2_ triangles that were clearly visible to the naked eye, which can support subsequent device fabrication and applications in various fields.

## Figures and Tables

**Table 1:** Growth morphology and related control factors.

	Triangular	Hexagonal	Large-Area
WSe_2_	31.8/10	32.4/8.5	32.2/10
MoSe_2_	32.1/15.5	32.7/11	32.3/15

## Data Availability

Data are available in a publicly accessible repository that does not issue DOIs or upon request from the corresponding author.

## Supplementary Materials

The following supporting information can be downloaded at: https://www.mdpi.com/article/10.3390/ma16134795/s1, Table S1: Current status of CVD method for growing two-dimensional materials as demonstrated in recent years; Figure S1: Growth of WSe_2_ crystals at different carrier gas flow rates; Figure S2: Growth of WSe_2_ with different distances between the precursors and the substrate; Figure S3: Images of large-size WSe_2_ grown on silicon substrate taken with a camera and WSe_2_ optical microscope images at 5× magnification; Figure S4: Full XPS spectrum. References [59,60,61,62,63,64,65,66,67,68,69,70,71,72,73,74] are cited in the supplementary materials.
