# Supplementary material for: Precise Construction and Growth of Submillimeter Two-Dimensional WSe2 and MoSe2 Monolayers
Source: Materials (Basel). 2023 Jul 3;16(13):4795. doi: 10.3390/ma16134795 (PMC10342866; doi:10.3390/ma16134795)
Supplement: Supplementary file 1 [file materials-16-04795-s001.zip › materials-2462157-supplementary.pdf]

## Supporting Information

# Precise Construction and Growth of Submillimeter Two-Dimensional $WSe_2$ and $MoSe_2$ Monolayers

**Yuqing Li [1], Yuyan Zhao [2], Xiaoqian Wang [1], Wanli Liu [1], Jiazhen He [1], Xuemin Luo [1], Jinfeng Liu [1] and Yong Liu [1,*]**

[1] International School of Materials Science and Engineering (ISMSE), State Key Laboratory of Advanced Technology for Materials Synthesis and Processing, Wuhan University of Technology, Wuhan 430070, China; lyqlosyimi@163.com (Y.L.); 303568@whut.edu.cn (X.W.); liuwani97@163.com (W.L.)

[2] Southwest Institute of Technical Physics, Chengdu 610041, China; yuyan_zhao0514@163.com

* Correspondence: liuyong3873@whut.edu.cn

**Table S1.** Current status of CVD method for growing two-dimensional materials in recent years

| | Materials | Methods | Size/Thickness |
|---|---|---|---|
| Wang et al.[1] | $TiSe_2$ | the metal and non-metal precursors | $5 \times 10^5$ μm$^2$ film /4.5nm |
| Meng et al.[2] | $CrTe_2$ | Conventional growth methods | ~80μm/1.2-47.9nm |
| Li et al.[3] | $ReS_2$ | changing the growth substrate | 10μm/No data |
| Chubarov et al.[4] | $WS_2$ | | Wafer-scale film/0.92nm |
| Kang et al.[5] | $MoS_2$, $WS_2$ | the metal and non-metal precursors | Wafer-scale film/monolayer |
| Yu et al.[6] | $WSe_{2(1-x)}Te_{2x}$ | tuning the elemental compositions of the precursors | No data/monolayer |
| Liu et al.[7] | $W_xMo_{1-x}S_2$ | | 20μm/0.94nm |
| Wu et al.[8] | Bi2O2Se | | ~200μm /6.7nm |
| Hong et al.[9] | $MoSi_2N_4$ | | ~1cm/1.17nm |
| Wang et al.[10] | Graphene | space-confined growth | ~3mm/~1.26nm |
| Xu et al.[11] | | | ~0.6mm/No data |
| Zhou et al.[12] | 47 different atomically thin TMCs | assistance of molten salts | 2μm-1mm/ monolayer |
| Cui et al.[13] | $Cr_2S_3$ | | ~60μm /1.84nm |
| Zhang et al.[14] | CrSe | | 0.15mm/2.50nm |
| Yang et al.[15] | $MoS_2$ | | 400μm/0.77nm |
| Zeng et al.[16] | $PdSe_2$ | thermally assisted conversion | Wafer-scale film /1.2-20 nm |
| Zhou et al.[17] | $MoTe_2$ | | Wafer-scale film /3.1nm |
| Yu et al.[18] | $MoS_2$ | | 1mm/8nm |

We show the parameter tuning process with the triangular $WSe_2$ as a representative. There is no such probing process in the already experienced $MoSe_2$ growth process. The optical microscope images shown are not the complete experimental process. They are only some representative experimental data selected to show the process of continuous sample refinement during parameter tuning.

As shown in Figure S1a, when the flow rate of the $H_2$/Ar was 30.7 sccm, the grown sample was $WSe_2$ in many bulk agglomerated states. The carrier gas flow rate is not sufficient to transport the sublimated selenium powder to the central heating zone of the furnace cavity to react with the precursor, and simultaneously, the intermediate state products are not effectively formed, and the nucleation points of $WSe_2$ are difficult to obtain, making the growth difficult. The carrier gas flow rate was increased to 31.2 sccm, as shown in Figure S1b. At this time, the number of nucleation points was sufficient and some small triangles started to be generated on the substrate. When the carrier gas flow rate is further increased to 31.8 sccm, as in Figure S1c, there is a significant increase in the size of the product on the substrate. When the reaction gas flow rate reaches 33 sccm, the triangles are no longer present on the substrate. Hydrogen promotes growth, but also reacts with $WSe_2$, leading to corrosion (Figure S1d). The corrosion increases with increasing load.

At a distance of 15 mm, the transition phase $WO_{3-x}$ is far from the substrate, and the concentration reaching the substrate is not sufficient for effective growth of the film. Therefore, it tends to grow as bulk particles, as shown in Figure S2a. When the distance is adjusted to 11.5 mm (Figure S2b), triangular monolayer $WSe_2$ films can be clearly observed, and the size can also reach about 50 μm. This indicates that the concentration of the transition phase $WO_{3-X}$ increases and helps to form monolayer films, allowing the films to grow effectively. By decreasing the distance again to 10 mm, as shown in Figure S2c, it can be seen that the film size continues to increase, indicating that the distance at this point is more suitable for the growth of large-size films. After decreasing the distance again to 8.5 mm, as shown in Figure S2d, only a very small amount of unshaped particles are present on the substrate, indicating that the close distance between the intermediate phase products and the substrate may have caused a high

concentration of deposits, preventing nucleation points formation.

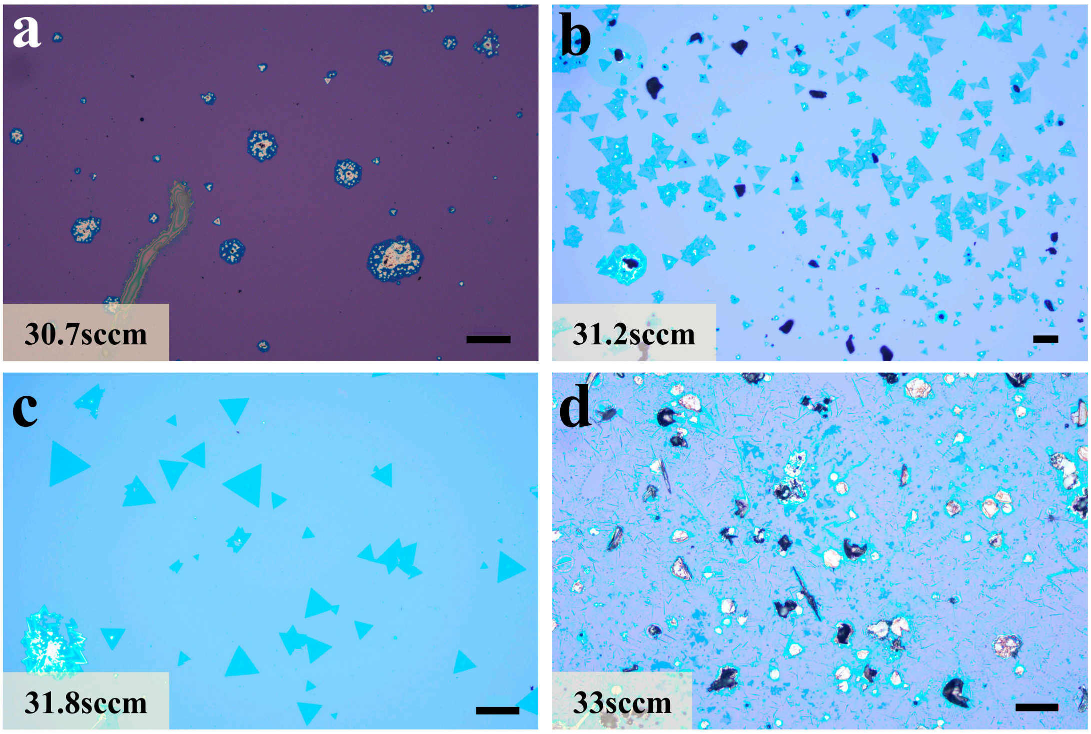


**Figure S1.** Growth of $WSe_2$ crystals at different carrier gas flow rates.(**a**,**b**) scale bar 50μm; (**c**,**d**) scale bar 100μm

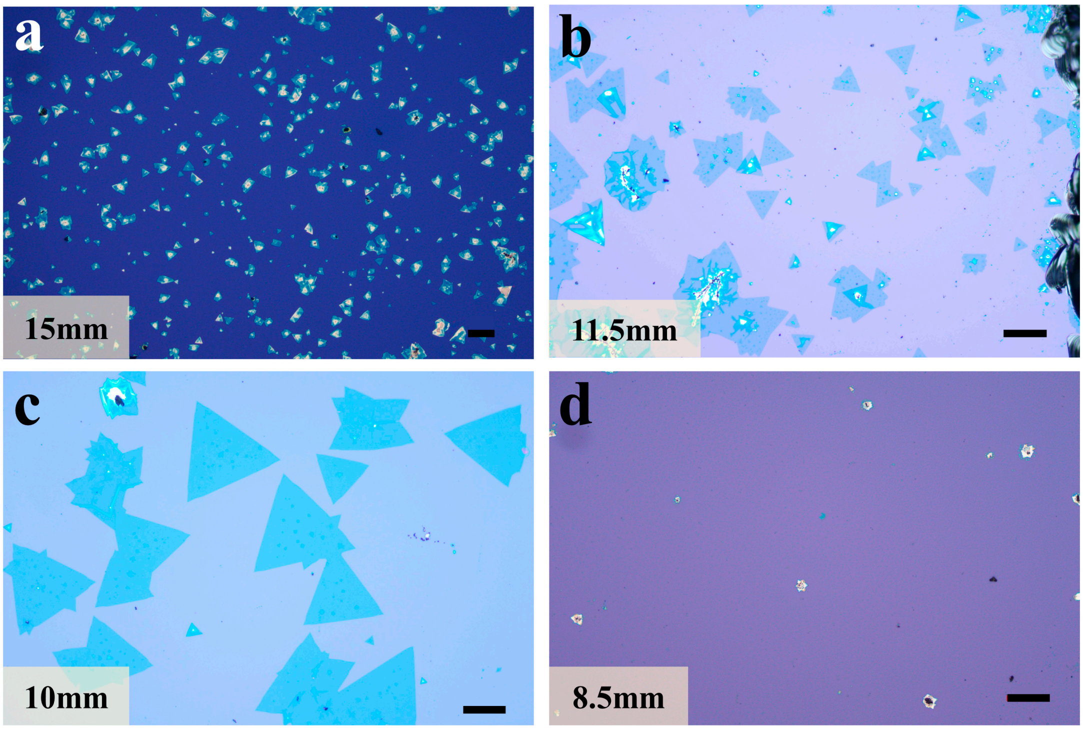


**Figure S2.** Growth of $WSe_2$ at different distances of precursors from the substrate. Scale bar 50μm

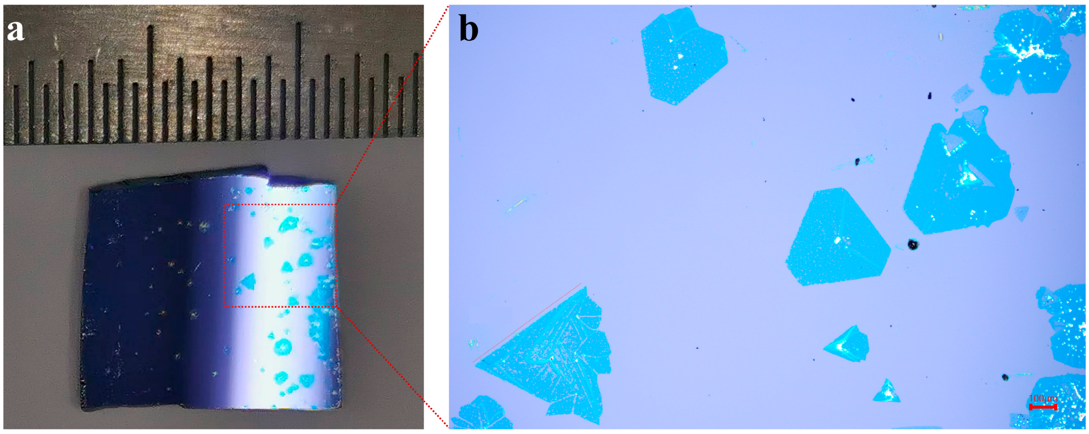


**Figure S3.** Large-size $WSe_2$ grown on silicon substrate taken with a camera and $WSe_2$ images at 5x optical microscope magnification. In this figure, $WSe_2$ appears as a triangular or truncated triangular multilayer shape. The area circled in red in (**a**) corresponds to the area in (**b**). Comparison with a standard scale shows that the maximum size reaches about 1 mm.

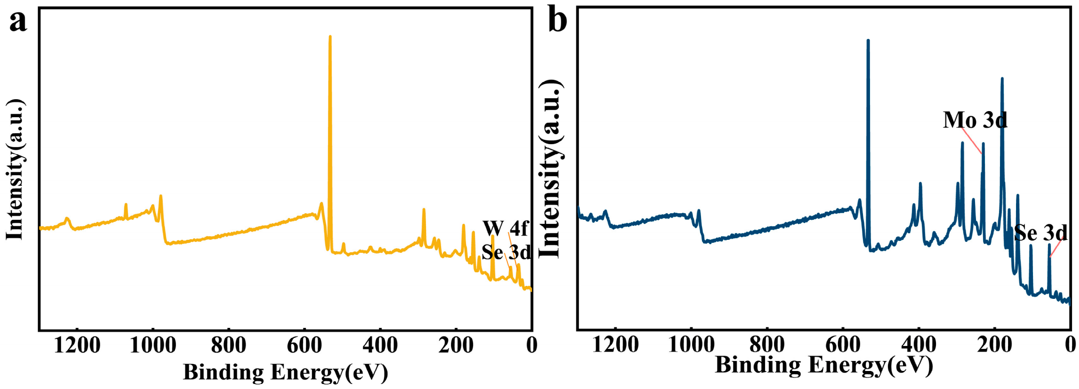


**Figure S4.** Full spectrum of XPS. (**a**) is $WSe_2$, and (**b**) is $MoSe_2$. The figure does not show signals of other metals and halides, which indicates the high chemical purity of the $XSe_2$ (X=W, Mo) crystals.

**References**


1. Wang, H.; Chen, Y.; Duchamp, M.; Zeng, Q. S.; Wang, X. W.; Tsang, S. H.; Li, H. L.; Jing, L.; Yu, T.; Teo, E. H. T.; Liu, Z., Large-Area Atomic Layers of the Charge-Density-Wave Conductor $TiSe_2$. *Adv. Mater.* **2018,** *30* (8).
2. Meng, L.; Zhou, Z.; Xu, M.; Yang, S.; Si, K.; Liu, L.; Wang, X.; Jiang, H.; Li, B.; Qin, P.; Zhang, P.; Wang, J.; Liu, Z.; Tang, P.; Ye, Y.; Zhou, W.; Bao, L.; Gao, H.-J.; Gong, Y., Anomalous thickness dependence of Curie temperature in air-stable two-dimensional ferromagnetic 1T-$CrTe_2$ grown by chemical vapor deposition. *Nat. Commun.* **2021,** *12* (1).
3. Li, X.; Dai, X.; Tang, D.; Wang, X.; Hong, J.; Chen, C.; Yang, Y.; Lu, J.; Zhu, J.; Lei, Z.; Suenaga, K.; Ding, F.; Xu, H., Realizing the Intrinsic Anisotropic Growth of 1T ' $ReS_2$ on Selected Au(101) Substrate toward Large-Scale Single Crystal Fabrication. *Adv. Funct. Mater.* **2021,** *31* (28).
4. Chubarov, M.; Choudhury, T. H.; Hickey, D. R.; Bachu, S.; Zhang, T.; Sebastian, A.; Bansal, A.; Zhu, H.; Trainor, N.; Das, S.; Terrones, M.; Alem, N.; Redwing, J. M., Wafer-Scale Epitaxial Growth of Unidirectional $WS_2$ Monolayers on Sapphire. *ACS Nano* **2021,** *15* (2), 2532-2541.
5. Kang, K.; Xie, S. E.; Huang, L. J.; Han, Y. M.; Huang, P. Y.; Mak, K. F.; Kim, C. J.; Muller, D.; Park, J., High-mobility three-atom-thick semiconducting films with wafer-scale homogeneity. *Nature* **2015,** *520* (7549), 656-660.
6. Yu, P.; Lin, J.; Sun, L.; Le, Q. L.; Yu, X.; Gao, G.; Hsu, C.-H.; Wu, D.; Chang, T.-R.; Zeng, Q.; Liu, F.; Wang, Q. J.; Jeng, H.-T.; Lin, H.; Trampert, A.; Shen, Z.; Suenaga, K.; Liu, Z., Metal-Semiconductor Phase-Transition in $WSe_{2(1-x)}Te_{2x}$ Monolayer. *Adv. Mater.* **2017,** *29* (4).
7. Liu, X.; Wu, J.; Yu, W.; Chen, L.; Huang, Z.; Jiang, H.; He, J.; Liu, Q.; Lu, Y.; Zhu, D.; Liu, W.; Cao, P.; Han, S.; Xiong, X.; Xu, W.; Ao, J.-P.; Ang, K.-W.; He, Z., Monolayer $W_xMo_{1-x}S_2$ Grown by Atmospheric Pressure Chemical Vapor Deposition: Bandgap Engineering and Field Effect Transistors. *Adv. Funct. Mater.* **2017,** *27* (13).
8. Wu, J.; Yuan, H.; Meng, M.; Chen, C.; Sun, Y.; Chen, Z.; Dang, W.; Tan, C.; Liu, Y.; Yin, J.; Zhou, Y.; Huang, S.; Xu, H. Q.; Cui, Y.; Hwang, H. Y.; Liu, Z.; Chen, Y.; Yan, B.; Peng, H., High electron mobility and quantum oscillations in non-encapsulated ultrathin semiconducting $Bi_2O_2Se$. *Nat. Nanotechnol.* **2017,** *12* (6), 530-+.
9. Hong, Y.-L.; Liu, Z.; Wang, L.; Zhou, T.; Ma, W.; Xu, C.; Feng, S.; Chen, L.; Chen, M.-L.; Sun, D.-M.; Chen, X.-Q.; Cheng, H.-M.; Ren, W., Chemical vapor deposition of layered two-dimensional $MoSi_2N_4$ materials. *Science* **2020,** *369* (6504), 670-+.
10. Wang, H.; Xu, X.; Li, J.; Lin, L.; Sun, L.; Sun, X.; Zhao, S.; Tan, C.; Chen, C.; Dang, W.; Ren, H.; Zhang, J.; Deng, B.; Koh, A. L.; Liao, L.; Kang, N.; Chen, Y.; Xu, H.; Ding, F.; Liu, K.; Peng, H.; Liu, Z., Surface Monocrystallization of Copper Foil for Fast Growth of Large Single-Crystal Graphene under Free Molecular Flow. *Adv. Mater.* **2016,** *28* (40), 8968-8974.
11. Xu, X.; Zhang, Z.; Qiu, L.; Zhuang, J.; Zhang, L.; Wang, H.; Liao, C.; Song, H.; Qiao, R.; Gao, P.; Hu, Z.; Liao, L.; Liao, Z.; Yu, D.; Wang, E.; Ding, F.; Peng, H.; Liu, K., Ultrafast growth of single-crystal graphene assisted by a continuous oxygen supply. *Nat. Nanotechnol.* **2016,** *11* (11), 930-935.
12. Zhou, J. D.; Lin, J. H.; Huang, X. W.; Zhou, Y.; Chen, Y.; Xia, J.; Wang, H.; Xie, Y.; Yu, H. M.; Lei, J. C.; Wu, D.; Liu, F. C.; Fu, Q. D.; Zeng, Q. S.; Hsu, C. H.; Yang, C. L.; Lu, L.; Yu, T.; Shen, Z. X.; Lin, H.; Yakobson, B. I.; Liu, Q.; Suenaga, K.; Liu, G. T.; Liu, Z., A library of

atomically thin metal chalcogenides. *Nature* **2018,** *556* (7701), 355-+.

13. Cui, F.; Zhao, X.; Xu, J.; Tang, B.; Shang, Q.; Shi, J.; Huan, Y.; Liao, J.; Chen, Q.; Hou, Y.; Zhang, Q.; Pennycook, S. J.; Zhang, Y., Controlled Growth and Thickness-Dependent Conduction-Type Transition of 2D Ferrimagnetic $Cr_2S_3$ Semiconductors. *Adv. Mater.* **2020,** *32* (4).
14. Zhang, Y.; Chu, J.; Yin, L.; Shifa, T. A.; Cheng, Z.; Cheng, R.; Wang, F.; Wen, Y.; Zhan, X.; Wang, Z.; He, J., Ultrathin Magnetic 2D Single-Crystal CrSe. *Adv. Mater.* **2019,** *31* (19).
15. Yang, P.; Zou, X.; Zhang, Z.; Hong, M.; Shi, J.; Chen, S.; Shu, J.; Zhao, L.; Jiang, S.; Zhou, X.; Huan, Y.; Xie, C.; Gao, P.; Chen, Q.; Zhang, Q.; Liu, Z.; Zhang, Y., Batch production of 6-inch uniform monolayer molybdenum disulfide catalyzed by sodium in glass. *Nat. Commun.* **2018,** *9*.
16. Zeng, L.-H.; Wu, D.; Lin, S.-H.; Xie, C.; Yuan, H.-Y.; Lu, W.; Lau, S. P.; Chai, Y.; Luo, L.-B.; Li, Z.-J.; Tsang, Y. H., Controlled Synthesis of 2D Palladium Diselenide for Sensitive Photodetector Applications. *Adv. Funct. Mater.* **2019,** *29* (1).
17. Zhou, L.; Zubair, A.; Wang, Z.; Zhang, X.; Ouyang, F.; Xu, K.; Fang, W.; Ueno, K.; Li, J.; Palacios, T.; Kong, J.; Dresselhaus, M. S., Synthesis of High-Quality Large-Area Homogenous 1T ' $MoTe_2$ from Chemical Vapor Deposition. *Adv. Mater.* **2016,** *28* (43), 9526-+.
18. Yu, F.; Liu, Q.; Gan, X.; Hu, M.; Zhang, T.; Li, C.; Kang, F.; Terrones, M.; Lv, R., Ultrasensitive Pressure Detection of Few-Layer $MoS_2$. *Adv. Mater.* **2017,** *29* (4).
